# 

**Published:** 1891-08

**Authors:** 


### CONTRIBUTORS TO VOLUME XXXI.

Barker, T. Ridgway, M. D. . . . . . . . . . . . . . . . . . . . . . Philadelphia.
Barnum, Eugene E., M. D. . . . . . . . . . . . . . . . . . . Lancaster C. H., Va.
Benedict, A. L., M. D. . . . . . . . . . . . . . . . . . . . . . . . . . Buffalo.
Brown, George L., M. D. . . . . . . . . . . . . . . . . . . . . . . . . Buffalo.
Clark, Edward, M. D. . . . . . . . . . . . . . . . . . . . . . . . . . Buffalo.
Crockett, Montgomery A., M. D. . . . . . . . . . . . . . . . . . . . Buffalo.
Cushing, Clinton, M. D. . . . . . . . . . . . . . . . . . . . . . San Francisco.
Dowd, J. Henry, M. D. . . . . . . . . . . . . . . . . . . . . . . . . . Buffalo.
Frederick, C. C., M. D. . . . . . . . . . . . . . . . . . . . . . . . . Buffalo.
Hayd, Herman E., M. D. . . . . . . . . . . . . . . . . . . . . . . . . Buffalo.
Himmelsbach, George A., M. D. . . . . . . . . . . . . . . . . . . . . Buffalo.
Hopkins, Henry R., M. D. . . . . . . . . . . . . . . . . . . . . . . . Buffalo.
Hubbell, Alvin A., M. D. . . . . . . . . . . . . . . . . . . . . . . . Buffalo.
Ingraham, Henry D., M. D. . . . . . . . . . . . . . . . . . . . . . . Buffalo.
Jacobson, Nathan., M. D. . . . . . . . . . . . . . . . . . . . . . . . Syracuse.
Keen, W. W., M. D. . . . . . . . . . . . . . . . . . . . . . . . . Philadelphia.
Krauss, William C., M. D. . . . . . . . . . . . . . . . . . . . . . . . Buffalo.
Lewis, Daniel, M. D. . . . . . . . . . . . . . . . . . . . . . . . . New York.
Lothrop, Thomas, M. D. . . . . . . . . . . . . . . . . . . . . . . . . Buffalo.
Manley, Thomas H., M. D. . . . . . . . . . . . . . . . . . . . . . . New York.
Mays, Thomas J., M. D. . . . . . . . . . . . . . . . . . . . . . . Philadelphia.
Miller, John A., Ph. D. . . . . . . . . . . . . . . . . . . . . . . . . Buffalo.
Morton, Thomas S. K., M. D. . . . . . . . . . . . . . . . . . . . Philadelphia.
Mynter, Herman, M. D. . . . . . . . . . . . . . . . . . . . . . . . . Buffalo.
O'Brien, E. C. W., M. D. . . . . . . . . . . . . . . . . . . . . . . . Buffalo.
Parmenter, John, M. D. . . . . . . . . . . . . . . . . . . . . . . . . Buffalo.
Pohlman, Julius, M. D. . . . . . . . . . . . . . . . . . . . . . . . . Buffalo.
Potter, Frank Hamilton, M. D. . . . . . . . . . . . . . . . . . . . . Buffalo.
Potter, William Warren, M. D. . . . . . . . . . . . . . . . . . . . . Buffalo.
Price, Joseph, M. D. . . . . . . . . . . . . . . . . . . . . . . . Philadelphia.
Putnam, James W., M. D. . . . . . . . . . . . . . . . . . . . . . . . Buffalo.
Riley, Henry A., A. B., LL. B. . . . . . . . . . . . . . . . . . . . . New York.
Rochester, DeLancey, M. D. . . . . . . . . . . . . . . . . . . . . . . Buffalo.
Roberts, John B., M. D. . . . . . . . . . . . . . . . . . . . . . . Philadelphia.
Sellstedt, Lars G., Esq. . . . . . . . . . . . . . . . . . . . . . . . Buffalo.
Thornbury, Frank J., M. D. . . . . . . . . . . . . . . . . . . . . . . Buffalo.
Weigel, Louis A., M. D. . . . . . . . . . . . . . . . . . . . . . . . Rochester.
Wende, Ernest, M. D. . . . . . . . . . . . . . . . . . . . . . . . . . Buffalo.
Werner, Marie B., M. D. . . . . . . . . . . . . . . . . . . . . . . Philadelphia.
Wright, Adam H., M. D. . . . . . . . . . . . . . . . . . . . . . . . . Toronto.
American Association of Obstetricians and Gynecologists.
American Orthopedic Association.
Buffalo Medical and Surgical Association.
Harlem Medical Association.
Medical Society of the County of Chautauqua.
Medical Society of the County of Erie.
Medical Society of the County of Niagara.
New York Academy of Medicine, Orthopedic Section.
Philadelphia County Medical Society.
Philadelphia Polyclinic Medical Society.

### LIST OF ILLUSTRATIONS.

PAGE.

CONTRIBUTIONS TO BRAIN SURGERY. (JOHN B. ROBERTS, M. D.)

Fig. 1.—Fractured Cranium, Showing Point where Trephine was Applied to Outer Surface . . . . . . . . . . . . . . . . . . . . . . . . . 395

Fig. 2.—Inner Surface of same Specimen . . . . . . . . . . . . . . . . . 395

Fig. 3.—Diagram Showing Relations of Brain Tumor . . . . . . . . . . 403

Fig. 4.—Diagram Showing Lateral View of Cerebral Convolution and Fissures, to aid in making description clear . . . . . . . . . . . . . . . 405

NEURO-TOPOGRAPHICAL BUST. (KRAUSS) . . . . . . . . . . . . . . . . . 494

NEW CLUB-FOOT SHOE. (PARK) . . . . . . . . . . . . . . . . . . . . . . . 361

NEW EAR SCISSORS. (HUBBELL) . . . . . . . . . . . . . . . . . . . . . . . 163

PLEURA-TROCAR. (HARTWIG) . . . . . . . . . . . . . . . . . . . . . . . . 495

POLYCLINIC OPHTHALMOSCOPE. (JACKSON) . . . . . . . . . . . . . . . . . 206

PORTRAIT OF DR. FRANK HAMILTON POTTER.

Facing p. . . . . . . . . . . . . . . . . . . . . . . . . . . . . . 53

PORTRAIT OF DR. R. P. BUSH.

Facing p. . . . . . . . . . . . . . . . . . . . . . . . . . . . . . 439

PORTRAIT OF WILLIAM DEAN HOWELLS.

Facing p. . . . . . . . . . . . . . . . . . . . . . . . . . . . . . 641

POZZI, TREATISE ON GYNECOLOGY.

Fig. 1.—(151). Enucleation of Interstitial Myoma . . . . . . . . . . 631

Fig. 2.—(255). Gastro-hysteropexy . . . . . . . . . . . . . . . . . 631

STAMPS FOR RECORDING CASES.

Chest and Abdomen . . . . . . . . . . . . . . . . . . . . . . . . . 253

Gynecological . . . . . . . . . . . . . . . . . . . . . . . . . . . 254

Dr. Jos. Eastman, of Indianapolis, has received the degree of LL. D. from the Wabash College, of Indiana. The doctor is a genial gentleman of deservedly high standing in the profession, and we congratulate him on this high mark of esteem bestowed upon him by those who are in a position to know his worth.—*Lancet-Clinic.*

### Society Meetings.

The American Society of Microscopists.— The American Society of Microscopists will hold its fourteenth annual meeting in Washington, D. C., August 10 to 14, 1891.

The Society is composed of professionals and amateurs. The proceedings are extremely interesting, for, in the working sessions, experts give demonstrations of their lines of study; in the evenings exhibits are made of special subjects, and of anything beautiful or instructive any member or exhibitor may desire to display.

Officers — *President*, Frank L. James, St. Louis, Mo.; *Secretary*, W. H. Seaman, Washington, D. C.; *Treasurer*, A. C. Mellor, Pittsburgh, Pa.

American Neurological Association.—The following preliminary programme of papers to be read at the seventeenth annual meeting, to be held at Washington, D. C., September 22, 23, and 24, 1891:

A Case of Polio-Myelitis Anterior — death on twelfth day, with the account of the Microscopic Examination made by Dr. C. W. Burr, by Dr. Wharton Sinkler, of Philadelphia; A Contribution to the Therapeutics of Polio-Myelitis, by Dr. V. P. Gibney, of New York; Polio-Myelitis Acuta Adultorum, by Dr. Wm. C. Krauss, of Buffalo; Double Athetosis, by Dr. Wm. Osler, of Philadelphia; (1) Report, with specimens, of a Case cf Multiple Neuritis, (2) Case, with specimens, of Lead-Poisoning, followed by Locomotor Ataxia, terminating fatally by Uremia, (3) Exhibition of a Porencephalic Brain, (4) Report of Two Cases of Rare Myoclonic Disorder, by Dr. James Hendrie Lloyd, of Philadephia; (1) A Case of Thomsen's Disease, (2) Removal of a Neuroma, followed by Disappearance of Local Anæsthesia of fourteen years' standing, by Dr. G. L. Walton, of Boston; A Case of Trephining and Excision of the Cortex for Jacksonian Epilepsy, by Dr. W. W. Keen and Dr. Charles K. Mills, of Philadelphia; The Diagnosis of Certain Forms of Intra-Cranial Syphilis, by Dr. Landon Carter Gray, of New York; Recent Fissural Diagrams, by Dr. Burt G. Wilder, of Ithaca; Syphilis of the Spinal Cord, by Dr. Philip Zenner, of Cincinnati ; Lead-Poisoning, with Special Reference to the Spinal Cord and to Peripheral Nerve Lesions, by Dr. E. D. Fisher, of New York ; Triple Personality, by Dr. Irving C. Rosse, of Washington, D, C. ; A Case of Glosso-Labio-Laryngeal Paralysis, with the Presentation of Specimens, by Dr. Graeme M. Hammond, of New York ; The Virile Reflex in Relation to Clinical and Forensic Neurology, by Dr. C. C. Hughes, of St. Louis ; (1) Facial Hemi-hypertrophy, (2) Five Recent Cases of Brain Surgery, by Dr. William A. Hammond, of Washington, D. C. ; A Case of Tumor of the Cerebellum, in which Trephining was done for the Relief of Pressure, by Dr. Philip Coombs Knapp, of Boston ; Lithemia Considered in its Relation to Nervous Phenomena, by Dr. C. Eugene Riggs, of St. Paul.

---

American Dermatological Association.—The following is the programme of the fifteenth annual meeting, to be held at the Shoreham Hotel, Washington, D. C., September 22, 23, 24, and 25, 1891:

OFFICERS FOR 1891.

*President,* F. B. Greenough, M. D., of Boston ; *Vice-President,* L. N. Denslow, M. D., of St. Paul ; *Secretary and Treasurer,* George Thomas Jackson, M. D., of New York.

FIRST DAY, TUESDAY, SEPTEMBER 22D.

Business Meeting (with closed doors) at 9.30 A. M. ; Report of Council ; Nomination of Officers for the ensuing year ; Appointment of Auditing Committee ; Proposals for Active and Honorary Membership ; Miscellaneous Business.

Morning Session at 10.30 o'clock. — Report of Committee on Nomenclature, and discussion thereon.

PAPERS :

Dermatitis Hemostatica, by Dr. H. G. Klotz ; A Case of Lupus Erythematosus with Fatal Complications, by Dr. W. A. Hardaway ; Report of a Case of Universal Erythema Multiforme, with colored portrait and specimen, by Dr. L. A. Duhring ; An Unusual Case of Sarcoma involving the Skin of the Arm : Amputation : Recovery, by Dr. F. J. Shepherd ; Multiple Sarcomata : History of a Case showing Modification, and Amelioration of Symptoms with large Doses of Arsenic, by Dr. S. Sherwell.

SECOND DAY, WEDNESDAY, SEPTEMBER 23D.

Business Meeting (with closed doors) at 9.30 A. M. — Report of Treasurer and Auditing Committee ; Election of Officers ; Election of Active and Honorary Members ; Selection of Time and Place of next Meeting ; Miscellaneous Business.

Morning Session at 10.30 o'clock. — Report of Committee on Statistics.

PAPERS :

Discussion of Tuberculosis of the Skin :

Its Clinical Aspects and Relations, by Dr. J. C. White ; Its Pathology, by Dr. J. T. Bowen ; Its Treatment, by Dr. G. H. Fox ; Thirteen cases of Tuberculosis of the Skin, with their Treatment, by Dr. J. S. Howe ; A Case of Lichen Scrofulosorum, by Dr. J. Grindon ; Notes of a Visit to the Leper Hospital at San Remo, Italy, with Photographs, by Dr. L. A. Duhring.

THIRD DAY, THURSDAY, SEPTEMBER 24TH.

Morning Session at 9.30 o'clock.

PAPERS :

The Treatment of Alopecia Areata, by Dr. P. A. Morrow ; A Therapeutic Note on Alopecia Areata, by Dr. L. D. Bulkley ; Morphia Atrophia of Wilson, by Dr. R. W. Taylor ; The Treatment of Pruritus, by Dr. E. B. Bronson ; Prairie Itch, by Dr. L. N. Denslow ; Diseases of the Skin associated with Derangements of the Nervous System, by Dr. W. T. Corlett ; Treatment of Chronic Ringworm in an Institution for Boys, by Dr. L. A. Duhring.

FOURTH DAY, FRIDAY, SEPTEMBER 25TH.

Morning Session at 9.30 o'clock.

PAPERS :

Notes of a Case of Acute Dermatitis Exfoliativa, by Dr. J. E. Graham ; Note Relative to Pemphigus Vegetans, by Dr. J. N. Hyde ; A Study of Mycosis Fungoides, with Report of a Case, by H. W. Stelwagon and H. Leffingwell Hatch ; Lymphangioma Circumscriptum, with Report of a Case, by Dr. M. B. Hartzell ; Remarks on Carbuncle, with Report of a Peculiar Case, by Dr. H. G. Klotz ; Note on Erythema et Naevus Nuchae, by Dr. C. W. Allen ; A Case of Lichen Ruber, by Dr. J. Grindon ; The Personal Equation in Dermatology, by Dr. L. D. Bulkley :

Retirement of old officers and induction of those newly elected ; Adjournment.

### BOOKS AND PAMPHLETS RECEIVED.

The Genuine Works of Hippocrates, translated from the Greek, with a Preliminary Discourse and Annotations. By Francis Adams, LL. D., Surgeon. Octavo, 766 pages, extra muslin, gilt top, price, $5.00. New York: William Wood & Company.

A Clinical Text-Book of Medical Diagnosis for Physicians, based on the Most Recent Methods of Examination. By Oswald Vierordt, M.D., Professor of Medicine at the University of Heidelberg, formerly Privat-Docent of the University of Leipzig; later Professor of Medicine and Director of the Medical Polyclinic of Jena. Authorized translation from the second improved and enlarged German edition, with additions. By Francis H. Stuart, A. M., M. D., New York. With 178 illustrations, many of which are in colors. 8vo, pp. xvi.—700. Philadelphia: W. B. Saunders. 1891. Price, cloth, $4.00 net; sheep, $5.00 net.

The Surgical Treatment of Wounds and Obstruction of the Intestines. By Edward Martin, M. D., Instructor in Operative Surgery, University of Pennsylvania, Surgeon to the Howard Hospital, Assistant Surgeon to the University Hospital; and H. A. Hare, M. D., Professor of Therapeutics, Jefferson Medical College; Attending Physician to St. Agnes' Hospital. Fiske Fund Prize Dissertation. No. XL., 8vo, pp.—169. Philadelphia: W. B. Saunders. 1891. Price, cloth, $2.00 net.

Insomnia and its Therapeutics. By A. W. Macfarlane, M. D., Fellow of the Royal College of Physicians, Edinburgh; Fellow of the Royal Medical and Chirurgical Society of London; Examiner in Medical Jurisprudence in the University of Glasgow; Honorary Consulting Physician (late Physician) Kilmarnock Infirmary; Formerly Examiner in Medicine and Clinical Medicine in the University of Glasgow, etc., etc. (Reprinted from Wood's Medical and Surgical Monographs.) Octavo, 302 pages; muslin, $1.75. New York: William Wood & Company. 1891.

Practical Intestinal Surgery, Vol. I. By Fred. B. Robinson, B. S., M. D., Professor of Anatomy and Clinical Surgery, Toledo (Ohio) Medical College; *Physicians' Leisure Library.* Issued monthly. Detroit: George S. Davis. 1891. Price, cloth, 50 cents; paper, 25 cents.

Lectures on Tumors from a Clinical Standpoint. By John B. Hamilton, M. D., LL. D., Professor of Principles of Surgery and Clinical Surgery, Rush Medical College, Chicago, and in the Chicago Policlinic; formerly Supervising Surgeon-General U. S. Marine Hospital Service, etc., etc. For the use of students. *Physicians' Leisure Library.* Detroit: George S. Davis. 1891. Price, cloth, 50 cents; paper, 25 cents.

Annual Report of the Superintendent of Education of the City of Buffalo. By James F. Crooker. 1889-90. Buffalo: Haas & Klein. 1891.

Annual Report of the Board of Managers of the Buffalo Historical Society, January 13, 1891, and the Society Proceedings. Buffalo: Published by order of the Society. 1891.

Three Communications to the Tenth International Medical Congress. By Paul Gibier, M. D., Director of the New York Pasteur Institute. 1. A New Theory about Temperaments. 2. Peroxide of Hydrogen; Ozone—Their Antiseptic Properties. 3. On Hydrophobia.

Nineteenth Annual Report of the Board of Inspectors of the House of Correction of the City of Chicago, for the year 1890. Presented by Mark L. Crawford, Superintendent.

The Chair of Surgery in Rush Medical College. By N. Senn, M. D. Ph. D., Professor of Surgery in Rush Medical College; Attending Surgeon to the Presbyterian Hospital. Introductory Address before the Class, April 11, 1891. Reprint: *Journal of the American Medical Association.*

Ueber Schwangerschaft nach konservativer Ventrofixatio uteri retroflexi. Von M. Sänger. (Vortrag, gehalten in der Sitzung der Gesellschaft für Geburtshülfe zu Leipzig, vom 16. März 1891.) (Sonder-Abdruck aus dem Centralblatt für Gynäcologie, 1891. No. 16.)

A Knowledge of Time Requirement. A Plea for a More Rational System of Medical Legislation. By Young H. Bond, M. D., Dean of the Marion-Sims College of Medicine, St. Louis, Mo. Reprint: *The Weekly Medical Review,* April 25, 1891.

Thirtieth Annual Report of the Cincinnati Hospital to the Mayor of Cincinnati, for the fiscal year ending December 31, 1890.

Pepsin. A Review of the Pepsin Question. By Dr. Carl Frederick Witte. Reprint: *Notes on New Remedies,* February and March, 1891.

Hypertrophy of the Pharyngeal Tonsil. A Clinical Lecture delivered October 30, 1890. By E. Fletcher Ingals, A. M., M. D., Professor of Laryngology in Rush Medical College, Chicago, Ill. Reprint: *The Medical News,* March 21, 1891.

The Shurly-Gibbes Treatment of Tuberculosis. By E. Fletcher Ingals, A. M., M. D., Professor of Laryngology in Rush Medical College; Professor of Diseases of the Chest and Throat in the Woman's Medical College; and John Edwin Rhodes, A. M., M. D., Professor of Diseases of Chest and Throat Clinic, Woman's Medical College. Reprint: *The Chicago Medical Record*, April, 1891.

The Surgery of Cleft Palate. By M. B. Ricketts, M. D., Cincinnati, O. Read before the Mississippi Valley Dental Society. March, 1891. Reprint.

NOTICE TO CONTRIBUTORS.—We are glad to receive contributions from every one who knows anything of interest to the profession. Articles designed for publication in the JOURNAL should be handed in before the first day of the month. The Editors are not responsible for the views or opinions of contributors. All communications should be addressed to 284 FRANKLIN STREET, BUFFALO, N. Y.

